# Decrease in Available Soil Water Storage Capacity Reduces Vitality of Young Understorey European Beeches (*Fagus sylvatica* L.)—A Case Study from the Black Forest, Germany

**DOI:** 10.3390/plants2040676

**Published:** 2013-10-23

**Authors:** Tamalika Chakraborty, Somidh Saha, Albert Reif

**Affiliations:** 1 Chair of Vegetation Science, Faculty of Environment and Natural Resources, University of Freiburg, Tennenbacherstr. 4, Freiburg D-79085, Germany; E-Mail: albert.reif@waldbau.uni-freiburg.de; 2 Chair of Silviculture, Faculty of Environment and Natural Resources, University of Freiburg, Tennenbacherstr. 4, Freiburg D-79085, Germany; E-Mail: somidh.saha@waldbau.uni-freiburg.de

**Keywords:** water stress, available soil water storage capacity, crown dieback, above ground biomass, tree survivability, semi-natural forest, summer drought of 2003, basal area increment

## Abstract

Growth and survival of young European beech (*Fagus sylvatica* L.) is largely dependent on water availability. We quantified the influence of water stress (measured as Available Soil Water Storage Capacity or ASWSC) on vitality of young beech plants at a dry site. The study site was located in a semi-natural sessile oak (*Quercus petraea* (Mattuschka) Liebl.) stand adjacent to beech stands on a rocky gneiss outcrop in southwestern Germany. Plant vitality was measured as crown dieback and estimated by the percentage of dead above ground biomass. The magnitude of crown dieback was recorded in different vertical parts of the crown. Biomass was calculated from the harvested plants following allometric regression equations specifically developed for our study site. Stem discs from harvested plants were used for growth analysis. We found that soil depth up to bedrock and skeleton content significantly influenced ASWSC at the study site. A significant negative correlation between ASWSC and crown dieback was found. Highest rates of crown dieback were noticed in the middle and lower crown. The threshold of crown dieback as a function of drought stress for young beech plants was calculated for the first time in this study. This threshold of crown dieback was found to be 40% of above ground biomass. Beyond 40% crown dieback, plants eventually experienced complete mortality. In addition, we found that the extremely dry year of 2003 significantly hampered growth (basal area increment) of plants in dry plots (ASWSC < 61 mm) in the study area. Recovery in the plants’ radial growth after that drought year was significantly higher in less dry plots (ASWSC > 61 mm) than in dry plots. We concluded that a decrease in ASWSC impeded the vitality of young beech causing partial up to complete crown dieback in the study site.

## 1. Introduction

The reduction in precipitation and increase of temperature during the growing season in recent years indicates a higher frequency of periodic drought in Central Europe [1]. Climatic data of the last 50 years shows a changing pattern following a positive and negative trend for temperature and precipitation, respectively, in southern Germany [2]. Climate models predict that the frequency of severe summer drought will increase in southern Germany under the scenario of global change that can directly impact forests. For example, the severe summer drought of 2003 reduced the net primary production of beech forest and caused high mortality in the southern part of Central Europe [3]. Trees are more prone to die in dry sites due to water stress in very dry and hot years such as 2003 [4,5]. Water stress can occur due to depletion of soil water and causes damage to plants by inhibiting plant vitality with profound changes in growth and morphology [4,6,7]. Plants’ tolerance to drought varies between different species. Past experiments on water stress tolerance in beech plants were carried out under laboratory or greenhouse conditions and found an influence of drought and water availability on beech survival, height and diameter growth [8,9,10,11]. The majority of these studies revealed that water stress could significantly reduce growth of beech plants in controlled laboratory conditions and experimental trial plots. In this context, European beech trees (*Fagus sylvatica* L.) are expected to decline in dry forest sites in the future under the ongoing climate change [12]. However, detailed investigations on the influence of water stress on vitality of young beech plants in dry site conditions at semi-natural forests are rare [5,13,14]. 

Tree vitality is a complex phenomenon, which is difficult to quantify in forest stands. Therefore, indicators like crown dieback are commonly used to assess it [15]. So far studies on water stress impact in forest stands have commonly assessed crown dieback by morphological classification or defoliation measurement, which are inevitably qualitative in nature [5,16]. However, quantitative assessment of crown dieback in proportion of actual plant biomass has never been carried out in trees grown in forests. Such methods would help to determine the dieback threshold in trees under drought stress. In addition, such information could help scientists and forest managers to assess the carbon loss in forests due to drought induced tree mortality. This crown-dieback threshold in terms of biomass can act as morphological indicator for the plants’ internal system failure, which may lead to complete plant death. Therefore, quantification of tree mortality and information on dieback threshold is very important in order to gain knowledge about the success of beech survivability under dry, semi-natural conditions in unmanaged forests. This motivated the authors to develop a novel approach to quantitatively assess crown dieback in young European beech plants. We precisely calculated the proportion of living and dead individual tree biomass to quantify crown dieback.

Severe drought events such as the European summer drought of 2003 can significantly reduce growth in beech trees [17]. The magnitude of reduction in radial growth after severe drought and recovery aftermath may fluctuate along the gradient of soil water storage capacity. However, such assumptions had never been tested on young understorey beech plants grown in semi-natural forest condition where human management had been abandoned for many decades. The magnitude of crown dieback is thought to be higher in the upper crown of trees because of higher susceptibility of xylem cavitation in the upper portion of the crown, which eventually leads to failure in hydraulic conductivity and crown dieback [18,19]. In 2005, Kohler *et al*. first reported the highest amount of dieback in the upper portion of the crown after the severe drought year of 2003 among intermediate beech trees (average height 10 m) in southwestern Germany [5]. However, similar assumptions have not been tested on young understorey beech plants with shorter height (e.g., 30–250 cm). Survival of beech plants belonging to this height cohort is crucial for successful natural regeneration in semi-natural forests and maintenance of structural and compositional complexities in forests after abandonment of human management [20].

Therefore, this study aimed to quantify the impact of water stress (measured as soil available water storage capacity or ASWSC) on crown dieback and basal area increment of young beech plants in a semi-natural sessile oak (*Quercus petraea* (Mattuschka) Liebl.) stand adjacent to beech stands. The stand was a rocky gneiss outcrop located at the Schlossberg hill in the submontane zone of the Black Forest, southwestern Germany. We hypothesized that (1) decreasing ASWSC would increase crown dieback in beech trees; (2) crown dieback would be higher in the upper crown; and (3) growth recovery (increase in basal area increment) would be higher in plants grown in less dry plots (ASWSC > 61 mm) than dry plots (ASWSC < 61 mm) after the 2003 summer drought. In addition, we aimed to find the important soil parameters that control ASWSC in the study site.

## 2. Results

### 2.1. Available Soil Water Storage Capacity (ASWSC) in Stand

The average stand ASWSC combining all plots was 67.4 mm (standard error: 7.14). In the linear regression analysis we found that the depth of soil up to bedrock and the soil skeleton content were the most important factors that significantly controlled the soil water storage capacity in our study site. Interestingly, we did not find any significant relationship between slope and ASWSC. Increase in soil depth also increased ASWSC. However, an increase in soil skeleton content decreased ASWSC (see Table 1). 

**Table 1:** Results from linear regression analysis regarding the influence of soil physical properties and slope on available water storage capacity (ASWSC) (Model R^2^ = 0.961, F = 68.269, *df* = 6, *p* < 0.0001).

Model parameters	Model estimates	Standard error	*t*	*p* value
Model constant	−32.390	67.257	−0.482	0.636
**Dependent variable: ASWSC**				
**Independent variables **				
Soil depth up to bedrock	1.568	0.126	12.432	**0.000**
Slope of soil profiles	0.350	0.418	0.836	0.415
Sand	0.640	0.704	0.908	0.376
Clay	0.120	0.637	0.188	0.853
Silt	0.170	0.703	0.241	0.812
Soil skeleton content	−0.834	0.099	−8.421	**0.000**

### 2.2. Relation between ASWSC and Crown Dieback

**Figure 1:**
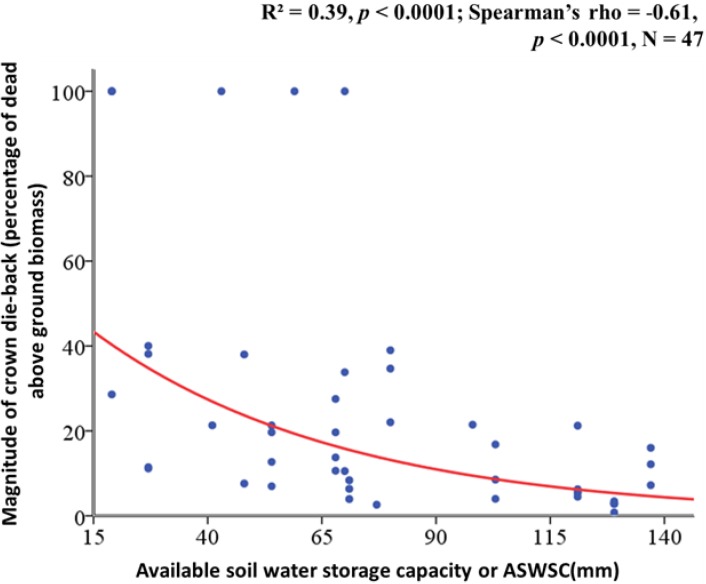
Relation between crown dieback, expressed by dead above ground biomass percentage and soil water stress calculated by the available soil water storage capacity (ASWSC in millimeters).

**Figure 2:**
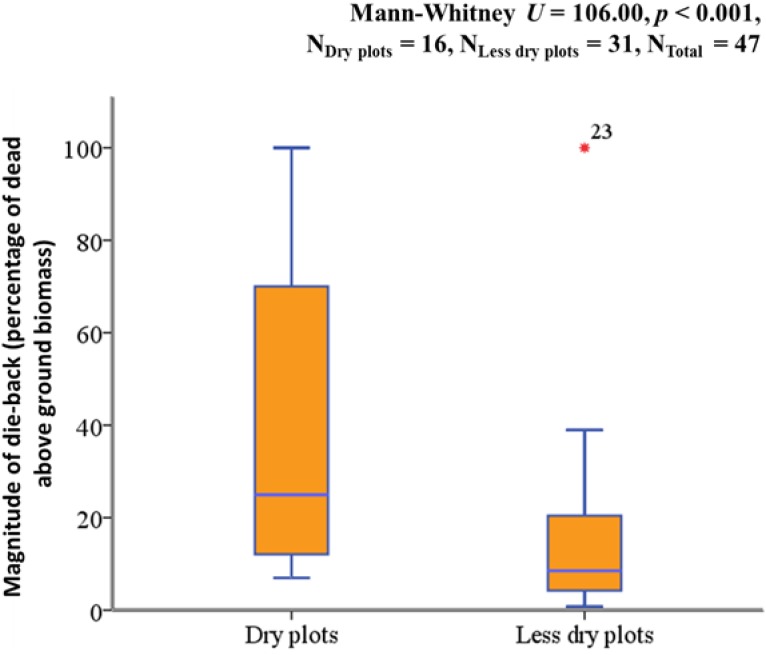
Magnitude of crown dieback (reported as median) was significantly higher among plants in dry plots (22%) than less dry plots (8%).

We found a significant and strong negative correlation between the magnitude of crown dieback and ASWSC (Spearman’s rho = −0.61, R^2^ = 0.39 *p* < 0.001) (Figure 1). This means that as the level of ASWSC increases, the crown dieback decreases in young beech plants. Crown dieback was significantly higher in dry plots (ASWSC < 61 mm) than in less dry plots (ASWSC > 61 mm) (Figure 2).

**Figure 3:**
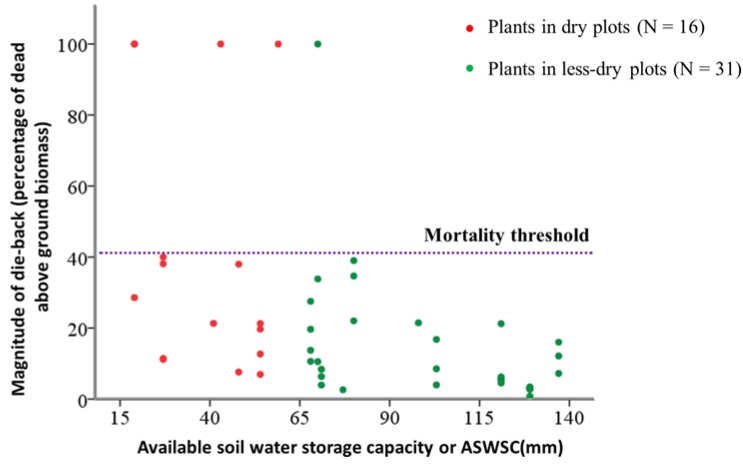
Mortality threshold of crown dieback, expressed by above ground dead biomass percentage for the young beeches in dry and less dry plots, expressed by available soil water storage capacity (ASWSC in millimeters). The figure shows the crown dieback threshold at 40%.

In this study, we found an interesting result regarding the crown dieback or mortality threshold in young beech plants. We did not find any single surviving plants in our total sample of 47 (42 live and five dead plants) that had more than 40% crown dieback. This means that irreversible damage had happened to the plant when dieback reached the threshold of 40%, which eventually lead to the death of the whole plant (Figure 3). The probability of whole plant death increased significantly for the plants which were found in dry plots (see Table 2). 

**Table 2:** Results from binary logistic regression, where a status plant (live or dead) was selected as dependent variable and ASWSC of plots (dry or less dry) was considered as independent variable (Log likelihood ratio value: 26.830, Cox and Snell R^2^ = 0.101, Nagelkerke R^2^ = 0.206, N = 47).

Parameters	Model estimate (β)	Standard error for model estimate	Wald Chi square	*df*	*p* value
Less dry plots *vs*. Dry plots	−2.303	1.169	3.879	1	0.0389
Model constant	3.401	1.017	11.195	1	0.0008

### 2.3. Partial Dieback in Different Crown Compartments

**Figure 4:**
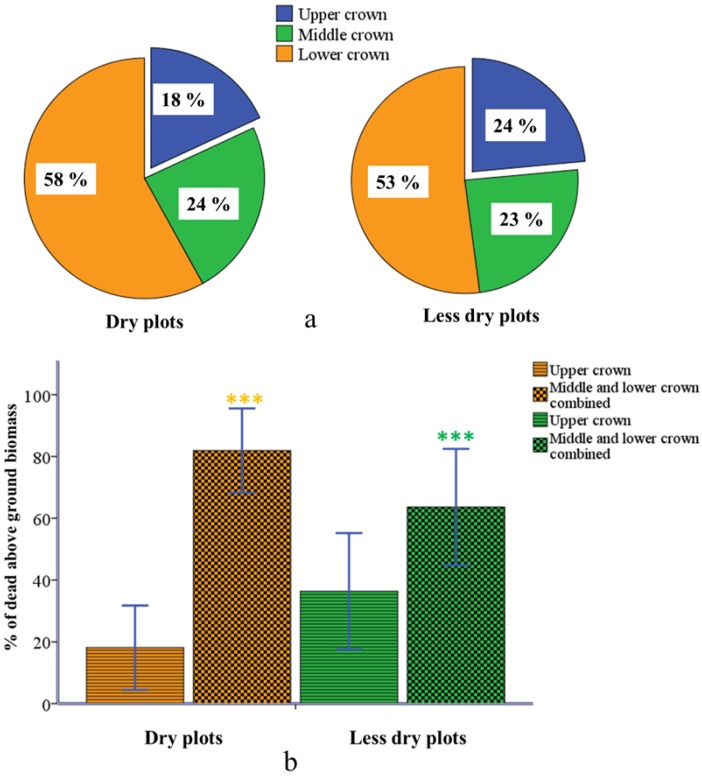
Magnitude of crown dieback in different vertical crown compartments of the dry and less dry plots. The pie-diagram (**a**) shows the percentage of crown dieback in plants from dry and less dry plots. The bar diagram (**b**) compares the percentages of crown dieback between upper crown and the rest of the crown (middle and lower combined) in dry and less dry plots. The thin bars represent the standard error of mean at a 95% confidence interval. Yellow and green asterisks show the significance level (*p* < 0.001).

The magnitude of crown dieback was different in the three vertical crown compartments in both dry and less dry plots (Figure 4a). The lower crown was affected by the highest dieback in both plot groups (dry plots: 58%; less dry plots: 53%). In both dry and less dry plots, crown dieback was significantly lower in the upper crown compared to the rest of the crown (dry plots: *t* = −5.144, *df* = 11, *p* < 0.001, N = 12; less dry plots: *t* = −5.981, *df* = 29, *p* < 0.001, N = 30) (Figure 4b).

### 2.4. Summer Drought of 2003 and Basal Area Increment

**Figure 5:**
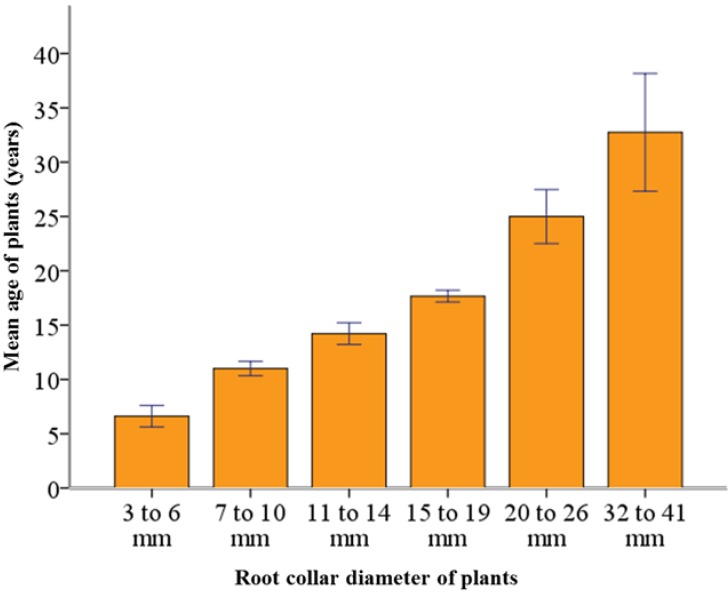
Age and root collar diameter of sampled beech plants (N = 42). Thin bars represent the standard error of mean at a 95% confidence interval.

The mean age of plants was 15 years and did not vary significantly between dry and less dry plots. The plants with root collar diameters of 3 to 6 mm had an average age of 6 years, whereas, the plants with 32 to 41 mm collar diameter had an average of 35 years (Figure 5). Out of 42 living plants, only four were born (two each in dry and less dry plots) in the drought year of 2003. Nevertheless, only one plant out of 42 (with >1 mm root collar diameter) was born after the drought year of 2003. That single plant was found in a less dry plot. 

**Figure 6:**
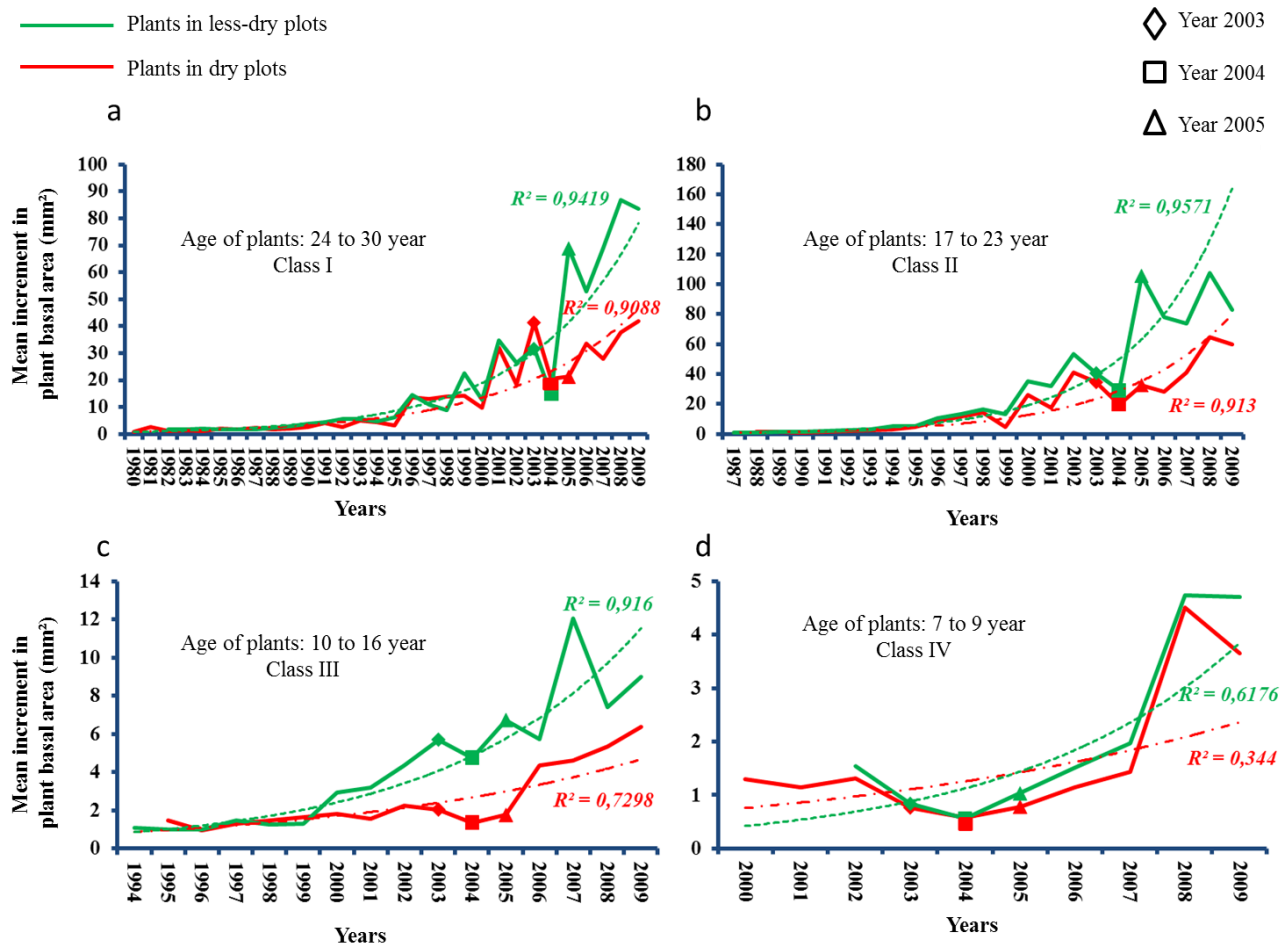
Trends in basal area increments in 42 beech plants in four different age classes (Class I = 24 to 30 year old, (**a**); Class II = 17 to 23 year old, (**b**); Class III = 10 to 16 year old, (**c**); and Class IV = 7 to 9 year old (**d**). The summer drought of 2003 was used as pointer year. Non-linear regression (after post hoc testing) with power function was used to report the strength in the growth trend.

Older plants (17 to 30 years old, age Class I and II) from dry and less dry plots had followed similar trends in basal area increment until 2003. However, after the 2003 summer drought, plants from less dry plots were following higher slopes in growth trajectory compared to plants from dry plots in age Class I and II. Since year of 1999, slope of growth trajectory was higher in plants from less dry plots than dry plots in age class III. However, this trend could not be observed in very young plants (7 to 9 years of age) (Figure 6). 

**Figure 7:**
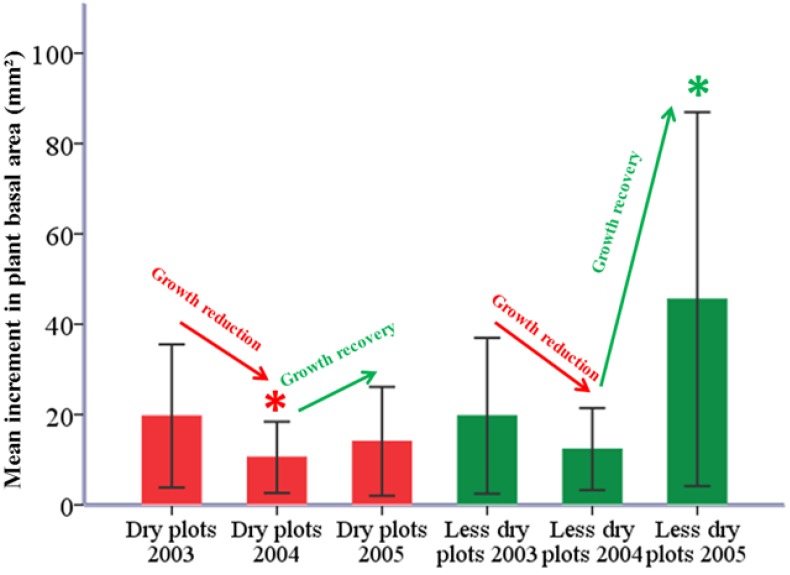
Comparisons of basal area increments between the years of 2003, 2004 and 2005 in dry (red) and less dry plots (green). Asterisks denote the level of significance (*p* < 0.05). Thin bars indicate the standard error of mean at a 95% confidence interval.

In both dry and less dry plots, there was a reduction of growth in 2004 compared to the previous drought year of 2003. This reduction was significant in dry plots but not in less dry plots (dry plots: *t* = 2.911, *df* = 11, *p* < 0.05, N = 12; less dry plots: *t* = 1.581, *df* = 11, *p* > 0.05, N = 12). However, recovery in basal area growth from 2004 to 2005 was significantly higher among plants located in less dry plots. This growth recovery was not significant for plants in dry plots (dry plots: *t* = 2.911, *df* = 11, *p* < 0.05, N = 12; less dry plots: *t* = 1.581, *df* = 11, *p* > 0.05, N = 12). Overall, this meant that the 2003 summer drought had a higher negative impact on plants which were located in dry plots than on plants in less dry plots (Figure 7).

## 3. Discussion

In a dry stand with a mean ASWSC of 67 mm, the magnitude of crown dieback significantly increased with a decrease in ASWSC. This is an agreement with our first hypothesis. This result corroborates previous studies which have found that under the prolong water stress, vitality decreases among trees in forests due to crown dieback [15]. We found that with an increase in soil water stress, the chance of survival of young beech plants diminished. This finding supports a previous study on oak trees, which showed that under chronic water stress in dry soil, the capability of young trees to overcome the stress or to survive diminished as their vitality decreased [21]. At a certain “*point of no return*”, irreversible damage would occur, which could eventually lead to permanent tree death [15]. Our results on crown dieback threshold show for the first time that this “*point of no return*” for young (30 cm to 250 cm height) beech plants is a 40% dieback in the crowns in terms of above ground biomass. 

Past studies have proven that forest stands with shallow soil and with high amounts of skeleton content have a high risk of water stress [12]. Water stress was found to be higher in such stands when they were located in the southwestern aspect as outcrop, hence, getting high solar exposure resulting in high evapotranspiration [14,16,22,23]. Gartner *et al*. working with various environmental, edaphic and topographical variables discovered that ASWSC would appear to be the most important limiting factor for beech survivability when it dropped below a threshold value of 68 mm at stand level [13]. The mean ASWSC in our stand was 67 mm, which supported this earlier assumption. In this study, five dead beech plants were sampled, which might cross the resistance threshold of 40% crown dieback when irreversible damage occurred as the cumulative effect of prolonged water stress, high solar irradiation and a catastrophic drought year of 2003 [24]. Based on the decline spiral model [25], severe drought years such as 2003 can operate as a trigger (“initiating factor”) that may ultimately lead to mortality in trees that are already under stress (by “predisposing factors” such as dry site conditions, here low ASWSC) and succumb to subsequent stem and root damage by biotic agents (“contributing factors” such as wood-boring insects, fungal pathogens *etc*.). Moreover, Pedersen (1998) argues that a “predisposing factor” and an “initiating factor” alone can lead to subsequent decline of tree vitality or to tree death. Vulnerability to tree death increased in this very dry stand, particularly after the extreme dry year of 2003 in the region, when the drought most likely acted as an initiating factor of mortality [5,21].

The magnitude of dieback varies in different crown compartments. The highest dieback, in both dry and less dry plots, was found in the middle and lower parts of the crown. Based on this finding, we rejected our second hypothesis. The middle and lower crown, which create the more shaded part of the whole crown, are less effective in photosynthesis in general. This may induce dieback patterns more severely affecting the middle and lower parts of the crown, which would be the first not to be supported in photosynthesis [26]. Our result is also corroborated with outcomes from past studies, which have shown that drought-induced cavitation and loss in hydraulic conductivity cause branch dieback that reduces the transpiration demand and thereby enabling the remaining shoots to maintain a favorable water balance [18,19,27]. Such an interpretation could be applied to many situations of growth decline in trees, e.g., where crown reductions or crown thinning resulting from branch abscission occurred after the onset of severe drought [28]. In addition to allowing dieback in lower parts of the crown under water stress, plants can regulate their root and shoot biomass and try to tune the root-shoot biomass ratio to maintain a specific correlation [29]. This strategy of root-shoot regulation in plants under prolonged water stress is called as “survival through dieback” [30]. In a dry year, parts of the tree dies back and only a few shoots survive, which are in balance with the root system. Branch dieback and the consequent reduction in whole plant leaf area are usually restricted to older and lateral twigs from the last order of branching. This would enable plants to adjust root-shoot ratios after drought induced decline in the root system, hence, crown dieback could be an acclimatization to drought stress [27,28,31]. Young beeches might choose a strategy of “survival through dieback” in this stand by allowing more branch dieback in lower parts of the crown and tuning root-shoot ratio [30]. However, they would not recover from this stress if they cross the threshold of 40% dieback in the crown in terms of above ground biomass.

Recovery in basal area increment after the drought year of 2003 was higher in plants from less dry plots than dry plots. This is an agreement with our third hypothesis. Our results prove that after a catastrophic drought event, plants growing in less dry plots have higher resistance (*i.e.*, lower growth reduction) and better resilience (*i.e.*, faster recovery in growth) than plants growing in dry plots. Differences in soil water availability explain why recovery of plants on dry plots was slower than less dry plots after the drought of 2003 [17]. The extreme drought of 2003 combined with the already existing water stress because of poor ASWSC at the study site had a strong negative impact on beech stem increment [17,32,33]. High temperatures accelerated the water deficiency from the soil, and this turned into a negative influence on the basal area increment of plants [15]. This problem became severe in dry plots as shown by our results. Czajkowski *et al*., who worked on the immediate impact of the 2003 summer drought on beech seedlings, reported that the plant water status during July and August of 2003 had a considerable effect on the relative increment of saplings in consecutive years [17]. This carry over effect observed by Czajkowski *et al*. corroborated our findings [17]. This phenomenon could be the consequence of high plasticity in broadleaved trees, which slowed down their growth to overcome the environmental stress in order to survive periods of poor growing conditions [34]. However, irreversible damage occurred if growth reduction led to crown dieback which in turn eventually forced the plants to cross the threshold. 

## 4. Experimental

### 4.1. Study Site and Sampling Design

**Figure 8:**
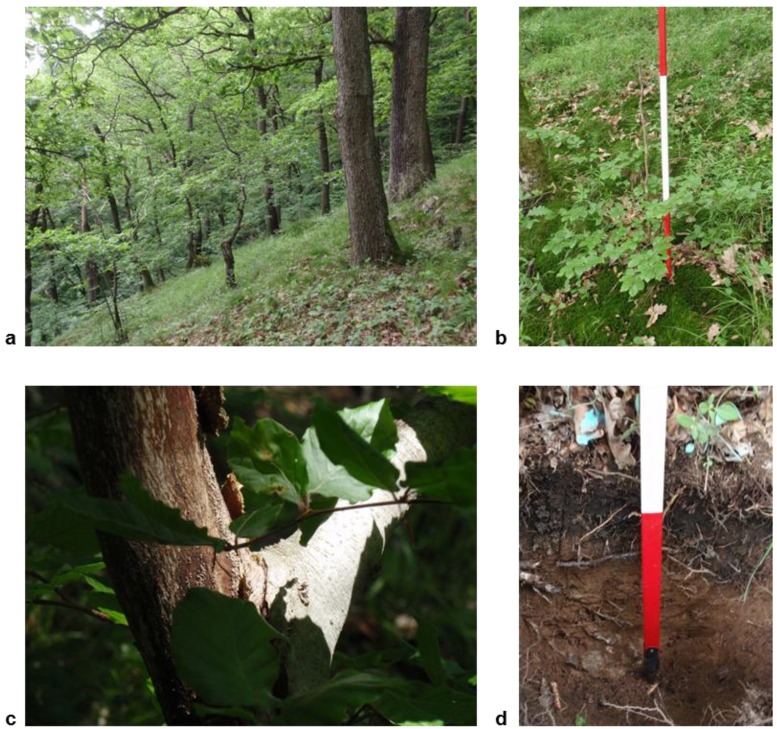
Study site at Schlossberg (**a**), partially dead standing young understorey beech plant (**b**), sampled beech plant with a dead branch on the left and a living branch on the right side (**c**), shallow soil profile at the study plot (**d**).

Data was collected in the summer of 2010 from a semi-natural sessile oak (*Quercus petraea*) stand (0.3 ha) with beech understory, surrounded by beech-dominated stands. The stand was located at Schlossberg hill in the Black Forest mountain region (near Freiburg city) in southwestern Germany (47°59'N, 07°51'E) in the submontane zone at 400 m above sea level. Since the medieval period the stand had been managed under coppice with a standard silvicultural system in order to fulfill the firewood demand of the city of Freiburg. However, since the end of the Second World War, the stand remained unmanaged, all commercial activity had been permanently suspended and the forest was declared as a protected forest. Only recreational activities are permitted in the forest area. The stand is exposed to a slope in the south-west; hence, it receives high solar radiation throughout the whole vegetation period. The mean annual temperature and precipitation are 10 °C and 930 mm, respectively. The sessile oak stand is on the slope of a rocky gneiss outcrop that has shallow soils with a sandy texture and a high skeleton content. The mean slope of the stand is calculated to be 33.33° (standard error: 4.67, N = 24). The soil contains a thin and uneven layer of humus or *hagerhumus*. Sometimes the mineral soil surface and bedrock (gneiss) are exposed, but in some places they are covered by moss (Figure 8).

A systematic sampling design was followed for data collection in order to capture the gradient of ASWSC. The stand was small in size, and, therefore, negligible variations in environmental conditions (exposition, irradiation and/or light condition) among plots were assumed. Nevertheless, as beech is a shade tolerant species, competition for light is not a major constraint for tree establishment and survivability under the oak canopy [35]. A 54 × 34 m rectangular borderline was selected inside the stand as the sampled area to establish the plots. A systematic grid of four rows and six vertical columns 10 m apart from one another was implemented in the rectangular design. Twenty-four circular sampling plots with a 2 m radius and a central point, with vertical and horizontal distances of 10 m from each other, were established [36]. 

### 4.2. Collection of Morphological Data

As we wanted to assess the impact of ASWSC on the establishment of European beech plants in the stand, we focused on young understorey beech plants with a height ranging from 30 to 250 cm. In the German forest inventory system, a height of 250 cm for broadleaved plants is regarded as the threshold for successful establishment of natural regeneration [37]. Morphological and growth parameters were recorded for 47 young beech plants (42 live and five dead) found in the 24 circular plots. For the 42 living plants, the diameter at root collar (5 cm above ground level) and the diameter of all living branches (>1.5 mm diameter) were measured, and the number of all living branches and annual shoots (<1.5 mm diameter) was counted. The crown was equally divided into three vertical parts, namely upper, middle and lower, starting from the first green branch as described by Kohler *et al*. [5]. Diameter and location of all dead branches in the three different crown compartments were recorded to quantify the dieback in different crown parts by using a biomass equation developed for the stand. Only the diameter at root collar (5 cm above ground level) was measured for the five dead beeches inside the plots. 

### 4.3. Collection of Soil Data and Quantification of ASWSC

#### 4.3.1. Collection of Soil Data from Forest Stand

One by one meter soil profiles were dug until they touched the bedrock in the center of all 24 sampling plots. The different horizons were designated; soil depth and percentage of soil skeleton of each mineral horizon were measured [38]. Colors of moist soil were recorded for each mineral horizon using Munsell^®^ Soil Colour Charts [39]. Soil samples were collected from each mineral horizon of each soil profile for texture analysis.

#### 4.3.2. Quantification of ASWSC

In this study, the magnitude of soil water stress was measured by ASWSC [13,40]. ASWSC is the maximum amount of fine earth available water, expressed in mm*/*10 cm. It is the difference between the water content values at field capacity and the permanent wilting point [41]. At first, collected samples were sieved using a 2 mm sieve to separate out gravels and stones. Then the soils were crushed using a mortar and pestle to mix the soil aggregates. Then sand, clay and silt fractions of the soil were determined by using an assessment of texture method developed by Food and Agriculture Organization of the United Nations, Working Group for Forest Site Classification of Germany, and Working Group on Soil Classification in Germany modified after Schack-Kirchner [42,43,44,45]. Both German and FAO texture classes were recorded. Soil organic matter content was calculated using the Munsell color of soil and soil texture class proposed by Schlichting *et al*. [39,46]. ASWSC was calculated in mm for each plot using soil horizon depth, soil skeleton content, soil texture, and soil organic matter content. ASWSC ranged from 19 to 137 mm for 24 plots. Twelve plots were classified as “dry” having 19 to 60 mm, and another 12 plots as “less dry” having 61 to 137 mm ASWSC, respectively (Table 3) [43]. Please see the detailed plot-wise calculation of ASWSC in the supplementary document namely “Quantification of ASWSC.xlsx” uploaded in Supplementary File 1. 

**Table 3:** ASWSC of 24 plots in the stand.

Dry plots			Less dry plots		
Serial no.	Plot no.	Plot ASWSC (mm)	Serial no.	Plot no.	Plot ASWSC (mm)
1	1/1	22	1	1/5	71
2	1/2	21	2	1/6	68
3	1/3	41	3	2/3	98
4	1/4	59	4	2/6	70
5	2/1	52	5	3/1	129
6	2/2	19	6	3/2	103
7	2/4	27	7	3/3	77
8	2/5	43	8	3/5	80
9	3/4	54	9	4/1	103
10	3/6	54	10	4/2	121
11	4/3	27	11	4/4	137
12	4/6	48	12	4/5	93
**average ASWSC 39**			**average ASWSC 96**		

### 4.4. Quantification of Crown Dieback

Crown dieback was measured as the percentage of above ground biomass. In this section we will describe the procedures and steps used to quantify crown dieback for beech plants in this study. 

#### 4.4.1. Development of Allometric Equations for above Ground Biomass from Harvested Plants

All 42 living beeches from the sample plots were harvested to develop allometric equations for quantitative biomass analysis (12 and 30 plants from dry and less dry plots, respectively). We created five diameter classes for branches based on the inventory (Section 4.2) as following: two to five millimeters, 5.1 to 8 mm, 8.1 to 11 mm, 11.1 to 18 mm and 18.1 to 41 mm. In total 80 branches (40 from the dry area and 40 from the less dry area) were used for biomass calculation and were selected from these diameter classes. All above ground living parts of the sampled plants including stem and perennial branches with bark, annual shoots with buds and leaves were collected. Three annual shoots from each of the three crown compartments were randomly collected from each harvested plant. 

Biomass of harvested samples was assessed by *ex situ* weighing after oven drying in the laboratory at the temperature of 105 °C to constant weight. All fractions weights were determined to ±0.1 g accuracy.

**Figure 9:**
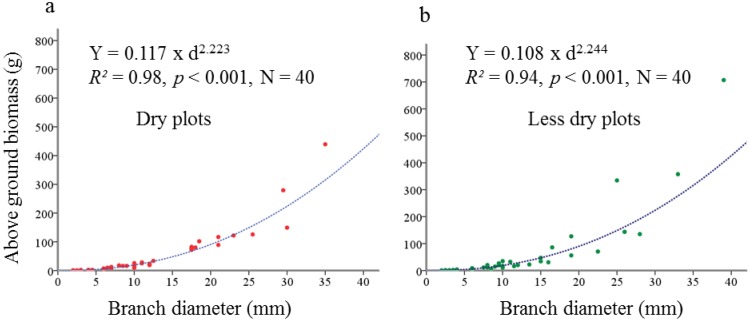
Regression model for biomass equation of the dry (**a**) and less dry plots (**b**). Biomass (Y) is modeled as the power function of stem/branch diameter (d). Biomass was measured in gram and diameter in millimeter (mm).

At first we performed *post hoc* model testing with 12 linear and nonlinear regression functions to develop equations for biomass. In those regression model tests, we used the weight of the branches as dependent variable and the diameter (mm) of the respective branches as independent variables. Finally, we found that non-linear regression with power function could explain the maximum amount of variation (resulted in the highest R^2^ value) in the data. This also supported a recent study that had shown that the biomass of almost all European broadleaved tree species followed a non-linear power function relationship with the diameter in young stages [47]. Finally, two non-linear regression models were formulated with biomass and diameter separately for both dry (Figure 9a) and less dry (Figure 9b) plots, using the biomass of stems and perennial shoots, according to the following formula:

Y = a × d^b^ (1)

where *Y* = biomass, *a* = coefficient constant, *b* = regression coefficient and *d* = stem/branch diameter. 

Mean of observed and modeled values from allometric equations were compared by *t*-test and showed no significant difference (Table 4). Hence, the allometric equations were accepted and used to calculate the biomass of branches in dry and less dry plots. 

**Table 4:** Results of model testing for biomass equations (Figure 9a,b and Figure 10) in dry and less dry plots.

Statistical tests	Dry plots	Less dry plots	Dead tree
*t*-test	*t*(39) = 0.72 *p* > 0.05	*t*(39) = 1.24 *p* > 0.05	*t*(41) = −0.22 *p* > 0.05

**Figure 10:**
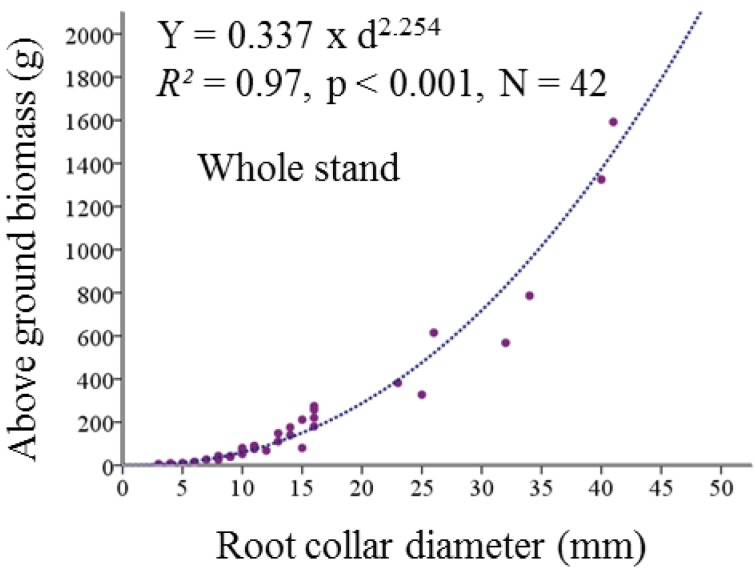
Regression model for above ground biomass equation of the dead trees. Above ground biomass (Y) is modeled as the power function of the diameter at root collar (d). Above ground biomass was measured in grams and the diameter at root collar in millimeters (mm).

The average biomass of annual shoots with buds and leaves was calculated separately for both dry and less dry plots. Biomass of total annual shoots of each tree was calculated by multiplying the average number with the total number of annual shoots recorded during the field study. The weights of annual shoots were summed up with the branch weight in order to get the total above ground biomass. In addition to the two above-mentioned equations for the branches, we also developed a biomass equation for living plants based on the root collar diameter (Figure 10). We also performed a *t*-test between modeled and observed values for this equation and did not find any significant difference (Table 4).

#### 4.4.2. Simulation of Dead above Ground Biomass

**Figure 11:**
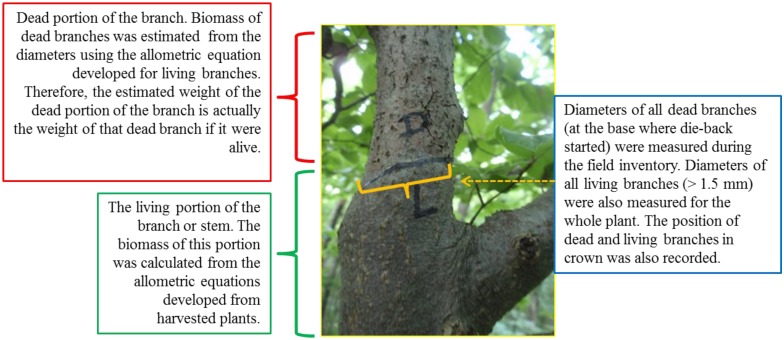
Schematic representation of the simulation method to quantify the crown dieback in terms of biomass.

Every living beech plant in our plots had dead branches or twigs. Those dead branches were either broken from the base or broken at a certain length from the base as a result of the branch shedding process. Practically, it was impossible to calculate the actual weight of that dead branch once it was green. We therefore developed a novel approach. We simulated the weight of a dead branch from the diameter recorded during field inventory. The diameter of such a dead branch was measured at the base where the mortality started and was still visible during the field inventory (Figure 11). Finally biomass of that dead branch was simulated from the allometric equations developed from the living branches of harvested plants specifically using the equation for dry and less dry plots. For a single tree, all dead branches were summed to calculate the total simulated weight of the dead branch. Similarly like the dead branches, above ground biomass of standing dead beech plants (five in total) were simulated from the regression model described in Figure 10.

#### 4.4.3. Calculation of the Proportion Dead above Ground Biomass (Quantitative Estimation of Crown-Dieback)

After we had calculated the total simulated dead above ground biomass and actual living biomass we made a simple proportion of these two biomass components in terms of percentages.

% of dead above ground biomass = (D/C) × 100
(2)

where *D* = dead simulated biomass of a tree, *C* = total above ground biomass of the tree. The increase in percentage values means an increase in crown dieback in terms of dead above ground biomass. For a tree without any mortality this proportion would be zero and for a totally dead tree this would be one hundred.

### 4.5. Tree-Ring Analysis for Calculation of Basal Area Increment (BAI) for the Growth Study

Tree-ring analysis was carried out using tree discs sampled at the root collar diameter at 5 cm above ground level. Stem discs from the 42 harvested plants were first used to calculate the age of the plants. After that, 24 plants (12 plants from dry plots and 12 from less dry plots) were selected to perform growth analysis. To minimize the confounding effect of the plant biological age on radial growth, we divided the plants in four age classes which were as follows: (1) Class I consisting of 24- to 30-year old plants; (2) Class II consisting of 17- to 23-year old plants; (3) Class III consisting of 10- to 16-year old plants; and (4) Class IV consisting of 7- to 9-years old plants. For every age class, we selected six plants (three plants in dry plots and three plants in less dry plots). 

**Figure 12:**
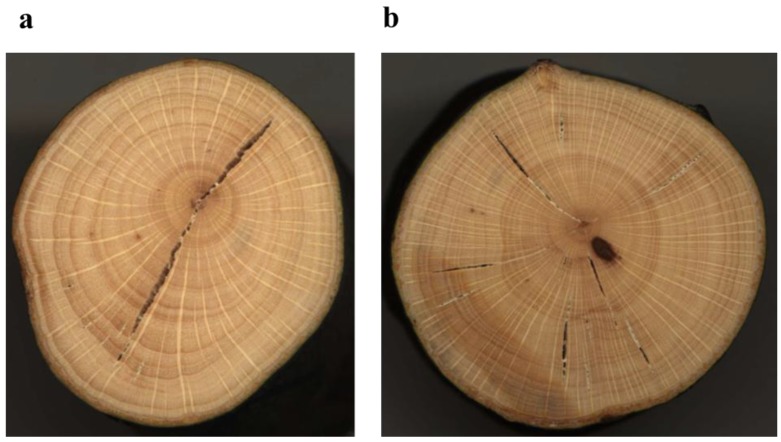
Stem discs for tree ring analysis, (**a**) 31-year-old beech plant with a root collar diameter of 33 mm from a less dry plot, (**b**) 42-year-old beech plant with a root collar diameter of 41 mm from a dry plot.

Samples were oven dried at 40 °C for 4 days and polished using successively finer sand paper to prepare them. Prepared samples were then scanned at 4,800 or 6,400 dpi using a LA1600+ scanner and saved as TIFF files (Figure 12). Ring boundaries were delimited manually on the images and ring widths were measured in four radial directions from the central pit to the periphery using the software WinDENDRO 2009 [48].

The ring width for each year was averaged from the four radial measurements to produce a final ring width series for each individual. Ring widths were converted into basal area increment (BAI), as this is more correlated with annual growth of the whole stem [49]. The following formula was used:

BAI = π(r^2^_n_ − r^2^_n-1_) (3)

where r = radius of the tree and n = the year of tree ring formation, π = 3.14. 

As our sample plants were very young, the intension of the third objective of this study was not to correlate annual temperature and rainfall variation with growth increment in beeches, but rather to investigate the immediate impact of the severe drought of 2003 on the growth of young beech plants. For this reason, we focused on 2003 as the pointer year, as it has been proven to be a year of nationwide drought. Therefore, we did not calculate a water balance calendar for climatic drought but instead used the literature as a proof of the 2003 summer drought [3,5,50]. The growth reduction and recovery of the plants were calculated separately for both dry and less dry plots. The growth reduction was the difference in basal area increment between the drought year of 2003 and the year 2004. Growth recovery was the increase in basal area increment from 2004 to 2005. 

### 4.6. Statistical Analyses

#### 4.6.1. First Hypothesis

At first we performed tests for normality (Kolomogorov-Smirnov test) on crown dieback (percentage of dead above ground biomass in 42 living plants and five completely dead but standing plants) and ASWSC of plots. We found that crown-dieback did not follow normal distribution, whereas, ASWSC followed normal distribution (see Normality Test 1 in Supplementary File 2). As data on crown dieback did not follow a normal distribution, we did non-parametric Spearman rank correlation tests to find the relationship between crown dieback and water stress (ASWSC). We did regression analysis to find whether crown-dieback could be explained by the variation in ASWSC. First we did *post hoc* regression analysis with 10 linear and nonlinear functions available in SPSS 20.0 [51], and selected an exponential function (non-linear) that explained the largest amount of variation (highest R^2^). To show whether the magnitude of crown dieback varied significantly between dry and less dry plots, a non-parametric Mann-Whitney *U* test was performed. In addition, we performed a binary logistic regression analysis to find out whether the probability of whole plant mortality was significantly higher in dry plots compared to less dry plots.

#### 4.6.2. Second Hypothesis

With this hypothesis we wanted to test whether the magnitude of crown dieback was significantly higher in the upper part of the crown compared to middle and lower parts. First, we used pie-diagrams to report the proportion of crown dieback (%) in upper, middle and lower parts of the crown in dry and less dry plots. Then we combined dieback from the middle and lower crown and compared this the to upper part by paired sample *t* test separately for dry and less dry plots. In this case we used parametric tests because dieback from the upper and remaining part of the crown was normally distributed in dry and less dry plots (see supplementary document uploaded in Supplementary File 2). 

#### 4.6.3. Third Hypothesis

We first plotted the mean annual basal area increment of plants from four age classes over time. In every age class, we fitted trend-lines separately for trees in dry and less dry plots. As exponential functions showed the highest R^2^ they were chosen to fit the trend-line in the basal area increment curve. Our intention by fitting trend-lines to the basal area increment curves was to check whether there was any difference in trends among plant growths over time in dry and less dry plots. To test whether the 2003 summer drought had a significant impact on growth reduction and recovery in plants, we did paired sample *t* tests between the mean basal area increments in 2003, 2004 and 2005. Mean basal area increments from both dry and less dry plots were normally distributed in those three years (see Supplementary File 2).

#### 4.6.4. Factors Influencing ASWSC

We wanted to find important soil and stand parameters (e.g., soil depth up to bed rock, slope of soil profiles, content of sand, clay, silt and soil skeleton), which had significant influence on the magnitude of ASWSC. The ASWSC was normally distributed (see Section 4.6.1). We also did normality tests for other parameters and found that all parameters followed a normal distribution (see Normality Test 4 in Supplementary File 2). 

As our data of the ASWSC, slope and other soil parameters were normally distributed we performed linear regression analysis. We used ASWSC as dependent variable and slope and other soil parameters as independent variable. All statistical analyses were done by SPSS v.20 [51].

## 5. Conclusions

We conclude that soil water stress (very low ASWSC) increases crown dieback, and hence, reduces tree vitality in a dry gneiss south-facing outcrop in the Black Forest. The threshold of crown dieback was found to be 40% in that semi-natural forest stand, which has not been under commercial management since the end of the Second World War. However, whether this threshold is consistent to a larger geographical scale in dry beech forest stands should be researched in future studies involving multiple sites. In addition, physiological processes behind this mortality threshold of young beech trees warrant further investigation.

In the future, an increased frequency of climatic drought might turn lethal for beeches on shallow soils where soil water stress is prevalent and could act as pre-disposing factor of tree mortality as an aftershock of one or multiple severe drought events [21]. Nevertheless, our study highlights the importance of causes and consequences of tree mortality in a dry site with shallow soil in a temperate forest.

Drought could lead to major compositional changes in plant species [52]. This in turn could initiate the development of new forest ecosystems due to new combinations of native and invasive exotic trees depending on the climatic and edaphic tolerances of seedlings [53,54]. This is a dynamic and long-term process which warrants further investigation, particularly near the distribution limits of beech forests on dry sites. 

## Supplementary Files

Supplementary File 1

Supplementary File 2
