# Supplementary material for: Decrease in Available Soil Water Storage Capacity Reduces Vitality of Young Understorey European Beeches (Fagus sylvatica L.)—A Case Study from the Black Forest, Germany
Source: Plants (Basel). 2013 Oct 23;2(4):676–98. doi: 10.3390/plants2040676 (PMC4844385; doi:10.3390/plants2040676)

Supplementary Material

Supplementary File 2

**Normality test 1.** Histograms and normality tests for crown dieback and ASWSC (Crown die-back: Kolomogorov-Smirnov Z = 1.726, *p* < 0.05, N = 47; ASWSC: Kolomogorov-Smirnov Z = 1.020, *p* > 0.05, N = 47).


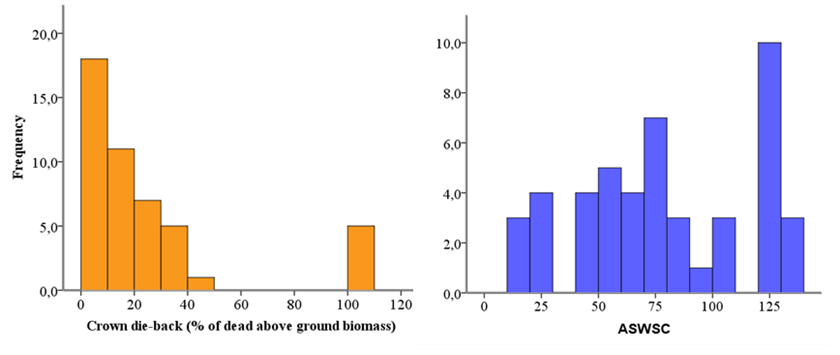


**Normality test 2.** Histograms and normality tests for crown dieback in crown compartments in dry and less dry plots. Upper crown in dry plots: Kolomogorov-Smirnov Z = 0.752, *p* > 0.05, N = 12; Less dry plots: Kolomogorov-Smirnov Z = 0.943, *p* > 0.05,
N = 30; remaining part of the crown in less dry plots: Kolomogorov-Smirnov Z = 1.042,
*p* > 0.05, N = 30).


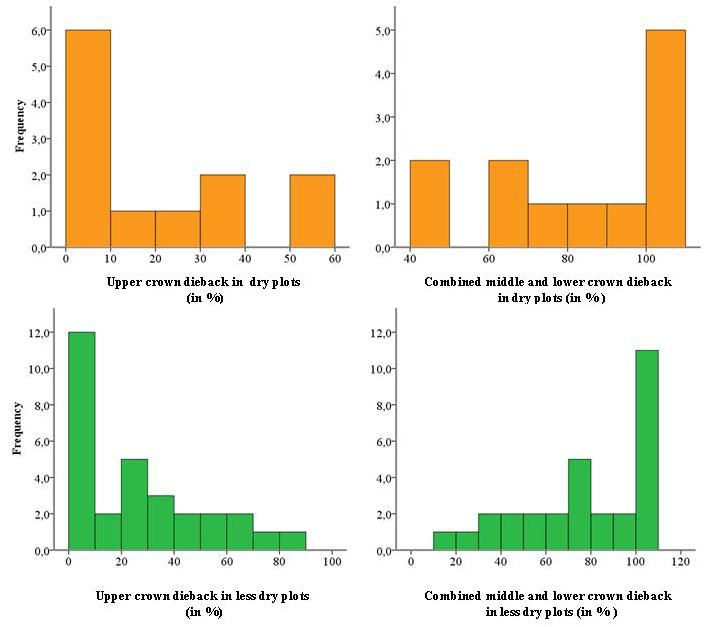


**Normality test 3.** Histograms and normality tests for basal area increments for dry and less dry plots in years 2004, 2004 and 2005.

|  | **Dry plots 2003** | **Dry plots 2004** | **Dry plots 2005** | **Less dry plots 2003** | **Less dry plots 2004** | **Less dry plots 2005** |
| --- | --- | --- | --- | --- | --- | --- |
| **Kolmogorov Smirnov Z** | 0.849 | 0.870 | 0.905 | 0.880 | 0.779 | 0.852 |
| ***p* value** | 0.467 | 0.436 | 0.385 | 0.421 | 0.578 | 0.462 |
| **N** | 12 | 12 | 12 | 12 | 12 | 12 |


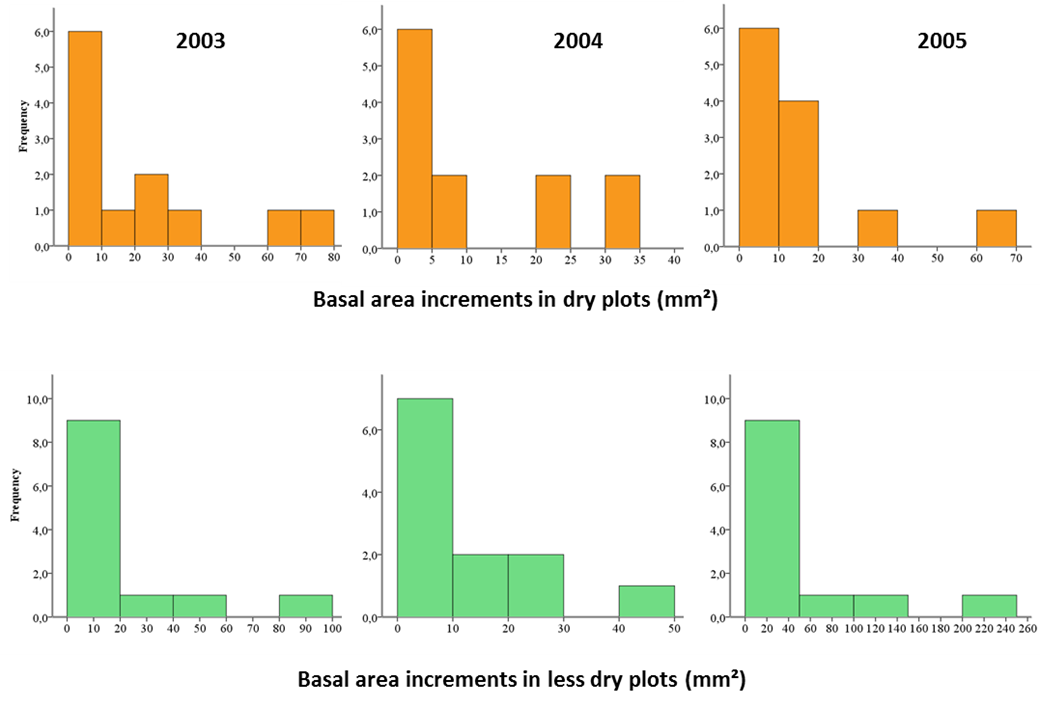


**Normality test 4.** Histograms and normality tests on soil parameters.

|  | **Soil depth up to bedrock** | **Slope of soil profiles** | **Sand** | **Clay** | **Silt** | **Soil skeleton content** |
| --- | --- | --- | --- | --- | --- | --- |
| **Kolmogorov-Smirnov Z** | 0.575 | 0.482 | 0.809 | 0.879 | 1.004 | 0.712 |
| ***p* value** | 0.896 | 0.974 | 0.529 | 0.422 | 0.265 | 0.691 |
| **N** | 24 | 24 | 24 | 24 | 24 | 24 |


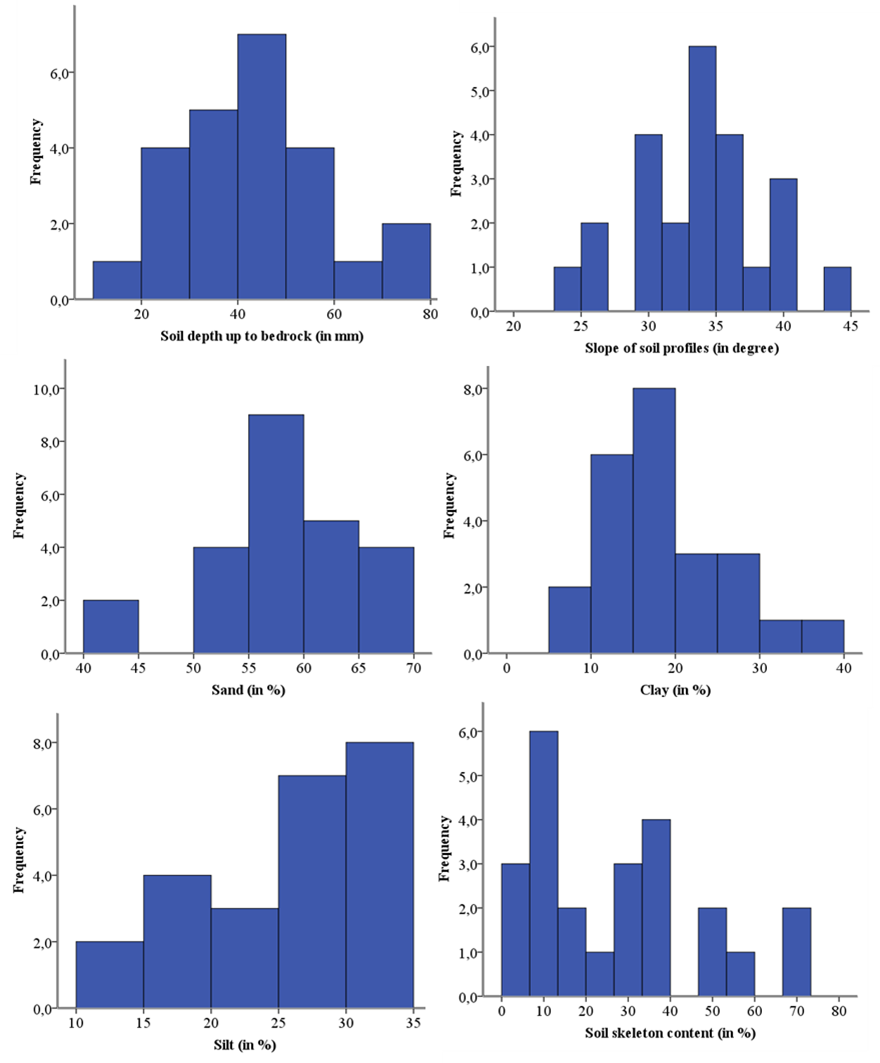

Supplement: Supplementary File 2 [file plants-02-00676-s002.docx]
